# CCR5 susceptibility to ligand-mediated down-modulation differs between human T lymphocytes and myeloid cells

**DOI:** 10.1189/jlb.2A0414-193RR

**Published:** 2015-05-08

**Authors:** James M. Fox, Richard Kasprowicz, Oliver Hartley, Nathalie Signoret

**Affiliations:** *Department of Biology and Hull York Medical School, Center for Immunology and Infection, University of York, York, United Kingdom; and ^†^Department of Pathology and Immunology, University of Geneva, Geneva, Switzerland

**Keywords:** chemokine receptor, Internalization, monocytes/macrophages, HIV-1

## Abstract

CCR5 is a chemokine receptor expressed on leukocytes and a coreceptor used by HIV-1 to enter CD4^+^ T lymphocytes and macrophages. Stimulation of CCR5 by chemokines triggers internalization of chemokine-bound CCR5 molecules in a process called down-modulation, which contributes to the anti-HIV activity of chemokines. Recent studies have shown that CCR5 conformational heterogeneity influences chemokine-CCR5 interactions and HIV-1 entry in transfected cells or activated CD4^+^ T lymphocytes. However, the effect of CCR5 conformations on other cell types and on the process of down-modulation remains unclear. We used mAbs, some already shown to detect distinct CCR5 conformations, to compare the behavior of CCR5 on in vitro generated human T cell blasts, monocytes and MDMs and CHO-CCR5 transfectants. All human cells express distinct antigenic forms of CCR5 not detected on CHO-CCR5 cells. The recognizable populations of CCR5 receptors exhibit different patterns of down-modulation on T lymphocytes compared with myeloid cells. On T cell blasts, CCR5 is recognized by all antibodies and undergoes rapid chemokine-mediated internalization, whereas on monocytes and MDMs, a pool of CCR5 molecules is recognized by a subset of antibodies and is not removed from the cell surface. We demonstrate that this cell surface–retained form of CCR5 responds to prolonged treatment with more-potent chemokine analogs and acts as an HIV-1 coreceptor. Our findings indicate that the regulation of CCR5 is highly specific to cell type and provide a potential explanation for the observation that native chemokines are less-effective HIV-entry inhibitors on macrophages compared with T lymphocytes.

## Abstract (precis)

The behavior of endogenous CCR5 receptors is influenced by the cellular context on primary human leukocytes.

## Introduction

CCR5 is a chemokine receptor mainly expressed on leukocytes, which mediates directed-cell migration and regulates cell activation during inflammation [1]. A GPCR, CCR5 responds to several agonistic chemokines, including CCL3, CCL3L1, CCL4, CCL5, and CCL8 [2]. Agonist binding triggers conformational changes in the receptor, activating the associated G protein and thereby initiating intracellular signals leading to a cellular response. CCR5 signaling is regulated, in part, through down-modulation, a process common to many GPCRs, in which activated receptors recruit β-arrestins that coordinate rapid endocytosis of the receptor [3].

CCR5 is also the principal coreceptor for the entry of HIV-1 into target cells, which include CD4^+^ T lymphocytes and macrophages [4–8]. Although CCR5-binding chemokines are capable of blocking HIV-1 entry into CCR5-transfected CD4^+^ cell lines and T lymphocytes [9–12], they are ineffective inhibitors of HIV-1 entry into macrophages [8, 13–18], a puzzling observation that has yet to be explained but has been attributed to differences in cellular factors [19–22]. Agonist-induced down-modulation is known to contribute to the anti-HIV activity of chemokines, as shown in vitro for CCR5 on transfected cells and activated CD4^+^ T cells [11, 23, 24] and suggested in vivo for CD4^+^ T cells [25]. Synthetic analogs of the CCR5 agonist, CCL5, have a greater capacity to induce receptor internalization, show increased anti-HIV-1 activity compared with CCL5 in vitro [26–28], and have good potency in vivo [29].

Conformational dynamics has a critical role in the functional regulation of GPCRs, which can elicit a range of different functional outcomes by adopting different conformations in response to ligands [30, 31]. These conformations differentially recruit cellular-effector proteins, notably heterotrimeric G proteins and β-arrestins, as well as neighboring GPCRs [32]. At the same time, these effector proteins, together with the lipid environment of a GPCR, are capable of affecting ligand affinity by allosterically modifying the receptor [33]. Hence, changing the cellular background in which a GPCR is expressed could be expected to have a profound effect on its functional behavior, a concept that we investigated in this study with CCR5.

Recent studies [34–36] have indicated that G protein-coupling could dynamically affect the conformational state of CCR5 in transfected cell lines, and the authors reported multiple conformations of CCR5 on in vitro activated CD4^+^ T cells defined by a set of mAbs, which differ in their capacity to interact with HIV-1 [37, 38]. However, those studies did not consider whether CCR5 conformational forms might exist on other cell types susceptible to HIV-1 infection or influence CCR5 response to chemokines in different cell backgrounds.

In this study, we used a series of mAbs directed against different conformational and linear cell surface epitopes of CCR5 to detect antigenically distinct conformations of CCR5 on in vitro activated T cell blasts, monocytes, or monocyte-derived macrophages and to measure the susceptibility of distinct populations of CCR5 to chemokine-mediated receptor down-modulation and HIV-1 entry inhibition. Our work reinforces the notion of CCR5 conformational heterogeneity, while revealing cell type- and ligand-specific sensitivity of the receptor to internalization and providing a possible explanation as to why macrophages are only poorly protected from HIV-1 infection by chemokines.

## MATERIALS AND METHODS

### Reagents and antibodies

Tissue-culture reagents, all secondary antibodies, and conjugated-streptavidin were purchased from Thermo Fisher Scientific (Paisley, Renfrewshire, United Kingdom). Other reagents were from Sigma-Aldrich (Gillingham, Dorset, United Kingdom), unless stated otherwise. All anti-CCR5 mAbs used were mouse anti-human: CTC5 (IgG1, MAB1802), CTC8 (IgG1, MAB1801), 45502 (IgG2b, MAB180), 45523 (IgG2b, MAB181), and 45531 (IgG2b, MAB182) were purchased from R&D Systems (Abingdon, Oxfordshire, United Kingdom), whereas 2D7 (IgG2a) was obtained from BD Pharmingen (Oxford, Oxfordshire, United Kingdom). Anti-CCR5 T21/8 (IgG1), anti-CD4 RPA-T4 (IgG1), and isotype-control antibodies (mouse IgG1, IgG2a, and IgG2b) were from eBioscience Ltd (Hatfield, Hertfordshire, United Kingdom). The mouse anti-CCR5 MC5 (IgG2a) [39, 40] from Dr. Matthias Mack (Department of Internal Medicine II, University of Regensburg Medical School, Regensburg, Germany) was biotinylated (biotin-MC5) using Pierce EZ-Link NHS-PEG solid-phase biotinylation kit (Thermo Fisher Scientific, Pittsburgh, PA, USA). Humanized anti-CD8 antibody (HIgG1 CD8) was from Dr. Herman Waldmann (Sir William Dunn School of Pathology, Oxford, Oxfordshire, United Kingdom). Alexa Fluor 488-, 594-, or 647-conjugated F(ab′)_2_ secondary antibodies [goat anti-mouse IgG (H+L), IgG1, or IgG2a] were purchased from Life Technologies (Warrington, Cheshire, United Kingdom). CCL4 and CCL5 were purchased from R&D Systems; CCL3, CCL8, and CCL3L1 were from PeproTech EC Ltd. (London, United Kingdom) and RANTES analogs were produced as described previously [41–43]. HIV-1_Ba-L_ (ARP118) was obtained from the Centre for AIDS reagents (National Institute for Biological Standards and Control, Ridge, Hers, United Kingdom), supported by the EC FP6/7 Europrise Network of Excellence, the NGIN Consortia, and the Bill and Melinda Gates Global HIV Research Cryorepository/Collaboration for AIDS Vaccine Discovery Project, and donated by Dr. Suzanne Gartner, Dr. Mikulas Popovic, and Dr. Robert Gallo [44].

### Cell culture and human blood cell isolation

DHFR-deficient CHO cells expressing human CCR5 (CHO-CCR5) were maintained as described previously [45]. PBMCs were isolated from anonymized human leukocyte cones (National Health Service Blood and Transplant Unit, Watford, Hertfordshire, United Kingdom), with monocytes and lymphocytes separated by adherence then cultured, differentiated, or activated in vitro (see Supplemental Fig. 1B). Monocytes were kept in complete RPMI 1640 medium (RPMI 1640 with 20 mM HEPES, 10% FBS, 100 units/ml penicillin, 0.1 mg/ml streptomycin and 2 mM l-glutamine) and were used for experiments up to 72 h after isolation, as described previously [40]. Monocyte-derived macrophages (MDMs) were generated by culturing monocytes for 9–15 d in complete RPMI 1640 medium with macrophage colony stimulating factor (50 ng/ml; PeproTech). T cell blasts were established from blood-isolated lymphocytes by 3 d of culture in complete RPMI 1640 medium containing phytohemagglutinin (5 µg/ml) followed by 9–15 d of culture with IL-2 (10 U/ml; PeproTech). Trypan blue exclusion was used to test cell viability. Monocytes, T cell blasts, and MDM cultures were assessed for purity, phenotyped, and CCR5 expression was characterized as reported in Supplemental Figs. 1 and 2, and only cells that showed CCR5 levels above the isotype control were used for experiments.

### Anti-CCR5 mAb binding, titration, and competition assays

CHO-CCR5 cells were detached from culture dishes with 10 mM EDTA in PBS, and MDMs were detached by gentle scraping. CHO-CCR5 and primary cells were resuspended at a density of 10^6^ cells/ml in ice-cold BM (RPMI 1640 without bicarbonate, with 10 mM HEPES and 2% BSA; pH 7.05) alone or containing 25 µg/ml human IgG, respectively. Cells dispensed in 96-well plates (5 × 10^4^ cells/well) were labeled for 1.5 h on ice with the indicated anti-CCR5 antibody (5 µg/ml) in BM, with or without 5 µg/ml human IgG. Samples were washed in ice-cold BM, fixed in PBS containing 3% formaldehyde (Polysciences, Inc., Eppelheim, Germany) and quenched, as described previously [46]. Fixed CHO-CCR5 and primary cells were stained for 1 h at RT in FACS buffer [PBS, 1%; FBS, 0.05%; sodium azide (NaN_3_)] with PE-conjugated or biotin-conjugated F(ab′)_2_ anti-mouse secondary antibody, respectively. Primary cells were finally stained with streptavidin-PE (1/500, BD Pharmingen). For titration experiments, cells were incubated with serial dilutions of the indicated mAb in BM for 90 min at 4°C, washed in ice-cold BM before fixation, quenching, and staining with an Alexa Fluor 647-conjugated anti-mouse secondary antibody. For competition assays, cells were labeled with serial dilutions of the CTC5 mAb in BM before addition of biotin-MC5 at a final concentration of 5 µg/ml and a further 90 min incubation. Samples were washed and stained with Alexa Fluor 647-conjugated streptavidin (1/500) for 1 h on ice before fixation. Cell-associated fluorescence was measured by flow cytometry using a FACSArray flow cytometer (BD Biosciences) from 10,000 accumulated events. Data were analyzed using the FACSArray or FlowJo 8.8.6 (Tree Star Inc., Ashland, OR, USA) software. Doublets were excluded, as described in Supplemental Fig. 2A and isotype-corrected MFI values were calculated by subtracting the fluorescence intensity of the relevant isotype control.

### CCR5 surface expression and down-modulation

Assays were performed as previously described [40]. In brief, 2 × 10^6^ cells/ml were incubated for 1 h or overnight (13–15 h) at 37°C in BM or tissue culture medium, alone or containing the indicated agonist (100 nM). All cells were then placed on ice, and blood-isolated cells were incubated for 20 min at 4°C with 25 µg/ml human IgG. All cells were labeled with anti-CCR5 mAbs (5 µg/ml) for 1.5 h at 4°C, washed, fixed, and quenched before being stained for 1 h at RT in FACS buffer with a PE-conjugated F(ab′)_2_ anti-mouse IgG secondary antibody. Cell-associated fluorescence was measured by flow cytometry as described above. Results are expressed as the percentage of surface expression by cells exposed to the medium alone (untreated) [(MFI treated/MFI untreated) × 100%] or the percentage of down-modulation {[1 − (MFI treated/MFI untreated)] × 100%], calculated from isotype-corrected MFI values.

### Immunofluorescence staining for microscopy

CHO-CCR5 cells and MDMs were seeded on coverslips at least 24 h before experiments, whereas monocytes and T cell blasts were treated in suspension. After treatment, samples were fixed in 3% formaldehyde and quenched, monocytes and T cell blasts were adhered onto poly-d-lysine-coated coverslips, and all samples were immunolabeled intact or saponin-permeabilized at RT, as described previously [39, 40] using the indicated anti-CCR5 mAb (5 µg/ml) and polyclonal Alexa Fluor-conjugated goat anti-mouse IgG (H+L), IgG1, or IgG2a, as indicated. DAPI (1 µg/ml, Life Technologies)-stained samples were mounted in Mowiol (Calbiochem, Merck Chemicals Ltd, Beeston, Nottingham, United Kingdom) and examined using a Zeiss (Welwyn Garden City, Hertfordshire, United Kingdom) LSM 510 confocal microscope. Images were analyzed using Zeiss LSM Image Browser software version 4, ImageJ software (National Institutes of Health, Bethesda, MD, USA), or Volocity software (PerkinElmer, Seer Green, Buckinghamshire, United Kingdom) and assembled using Adobe (San Jose, CA, USA) Photoshop CS6.

### Viral stock production and single-round infection assays

HIV-1_Ba-L_ virus was propagated by infecting 5 d differentiated MDMs with an MOI of 0.1. Supernatants were collected 9–20 d after infection, and aliquots taken to measure virus content by HIV-1 p24 ELISA (Aalto Bio Reagents Ltd, Dublin, Ireland). Frozen viral stocks were subsequently used in infection assays at an MOI of 1. MDMs were plated into 96-well plates (80,000 cells/well) and pretreated for 1 h on ice, with or without a 12.5 µg/ml concentration of the indicated antibody in the presence of 10 µg/ml human IgG-Fc fragments (Bethyl Laboratories, Inc., Montgomery, TX, USA), before adding the viral stock. Plates were transferred to a 37°C carbon dioxide incubator, and after overnight culture, supernatant was replaced with fresh medium, with or without the indicated antibody (12.5 µg/ml). Infection was assessed after 72 h of culture by HIV-1 p24 ELISA on cell supernatants.

### Statistics

Data from experiments performed in duplicate or triplicate on samples from *N* donors were analyzed with GraphPad Prism version 5.03 software using an ANOVA with the indicated multiple comparison posttest or a Student’s *t* test, where appropriate. Box and whisker plots show means (+ in boxes), medians (lines in boxes), 25th and 75th percentiles (boxes), and minimum or maximum values (whiskers). All other graphs show data expressed as means ± sd.

### Online supplemental material

Four supplemental figures describing the conditions used for in vitro cell culture and cell characterization (Supplemental Fig. 1), how we determined CCR5-specific expression on human blood cells (Supplemental Fig. 2), the method used to quantify the overlap of fluorescence between MC5 and CTC5 on MDMs (Supplemental Fig. 3), and the difference in CTC5 staining patterns after CCL5 treatment for T cell blasts and monocytes (Supplemental Fig. 4).

## RESULTS

### Anti-CCR5 antibodies used in the study

**Figure 1.**
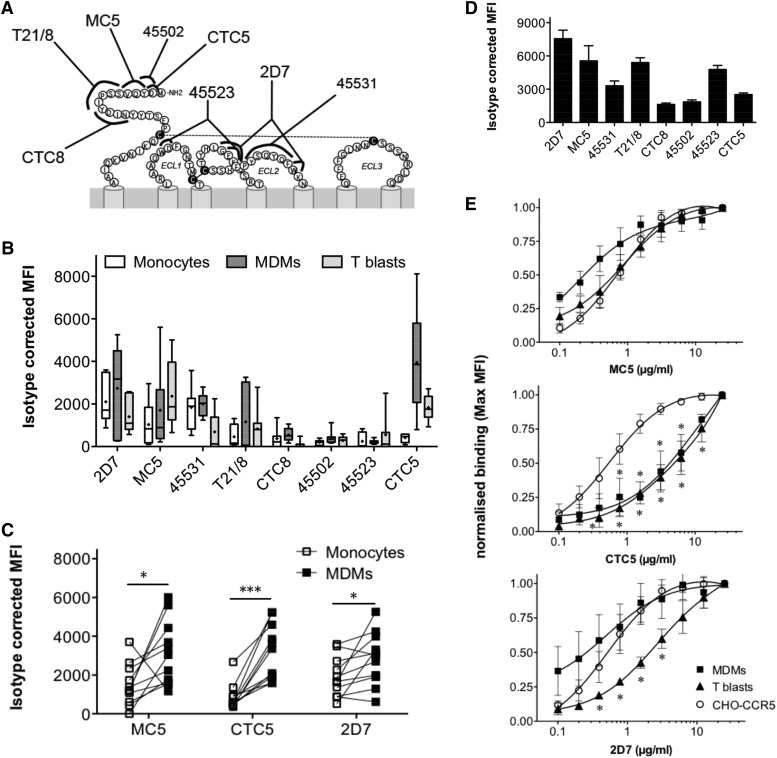
Anti-CCR5 mAb binding to human blood cells and CHO–CCR5 transfectants. (A) Diagram mapping the different CCR5 epitopes recognized by monoclonal antibodies used in our study. (B–C) Anti-CCR5 mAbs binding experiments performed on human monocytes, MDMs, and T cell blasts labeled live with a 5 µg/ml concentration of each anti-CCR5 mAb. Cell-bound antibodies were detected with biotin-conjugated secondary antibody followed by PE-streptavidin and cell-associated fluorescent signal measured by flow cytometry. (B) Box and whisker plots of isotype-corrected MFI values, showing the range of antibody-binding levels on cells derived from different donors (*N* = 7). (C) Cells derived from the same donors show a significant increase in MC5, CTC5, and 2D7 binding after differentiation of blood monocytes into MDMs (*N* = 11). *P ≤ 0.05 ***P ≤ 0.01 paired Student’s *t* test. (D) Like blood cells, CHO-CCR5 cells were labeled live with the different anti-CCR5 mAbs, but cell-bound antibodies were detected with a PE-conjugated secondary antibody; the graph plots the isotype-corrected MFI values (means ± sd) from a representative triplicate experiment. (E) Compared binding curves of each antibody for CHO-CCR5 cells, T cell blasts, and MDMs; results are normalized to the MFI of the highest antibody concentration and represent the means ± sd of *N* = 3 independent, triplicate experiments. *P < 0.05, 2-way ANOVA with Bonferroni posttest.

We used a panel of mouse anti-CCR5 mAbs to recognize different linear, multidomain, and conformation-dependent epitopes in the extracellular domain of CCR5 (**Fig. 1A**); some of which were previously used to study CCR5 conformations [35, 37]. Five of these mAbs (MC5, CTC5, 45502, T21/8, and CTC8) have been mapped to the N-terminal domain of CCR5 [47, 48]. MC5, 45502, and CTC5 recognize the first amino acid residues of CCR5 with expected overlapping binding sites, but only MC5 appears to recognize a linear epitope [37, 47, 49]. mAb 45523 recognizes residues within the first 2 extracellular loops (ECL1 and ECL2) and mAb 45531 in ECL2 [50, 51], whereas mAb 2D7, which is the most extensively studied anti-CCR5 antibody, binds an epitope in ECL2 that occludes the binding sites of chemokines and HIV-1 gp120 [50, 51].

### Detecting different antigenic forms of CCR5 on human blood cells and CHO-CCR5 cells

Monocytes, MDMs, and T cell blasts were derived from human peripheral blood-isolated mononuclear cells, phenotyped, and assessed for CCR5 cell surface expression using the mAbs MC5, CTC5, and 2D7 (see Supplemental Figs. 1 and 2). We looked at the representation of individual CCR5 epitopes on the different cell types by flow cytometry, labeling live cells on ice with 5 µg/ml of each anti-CCR5 mAb before fixation. Because blood cells expressed relatively low levels of CCR5 on their surface (estimated 1 × 10^3^ to 7 × 10^4^ ABS/cell [52]), cell-bound antibodies were detected after 2-step staining amplification with a biotinylated secondary antibody and PE-streptavidin. Figure 1B provides a qualitative overview of the variance in CCR5 epitope representation on cells from different individuals. Despite inherent donor variability that could result from CCR5 genetic polymorphisms [53], we observed broadly similar binding profiles across the different subsets of cells, with the exception of CTC5. The relative-binding levels of the antibody panel on T cell blasts was consistent with what was reported in an earlier study performed on activated CD4^+^ T lymphocytes [37]. In agreement with previously published work [54, 55], we found that MDM differentiation led to up-regulation of CCR5 cell surface expression, with a statistically significant increase in the binding signal of MC5, CTC5, and 2D7 between monocytes and MDMs from the same individual (Fig. 1C).

We compared these results with those obtained for CHO-CCR5 cells equally treated with a 5 µg/ml concentration of each anti-CCR5 mAb. With CHO-CCR5 cells having considerably more CCR5 molecules on their surface [3.4 × 10^5^ABS/cells; data not shown] cell-bound mAbs were revealed by direct staining with a PE-conjugated secondary antibody (Fig. 1D). As for blood cells, the CTC8 and 45502 mAbs showed reduced binding relative to 2D7, MC5, 45531, and T21/8 on CHO-CCR5 cells, but the notable difference to the profile obtained on monocytes, MDMs, and T cell blasts was the prominent representation of the 45523 epitope (Fig. 1B and D). These findings suggest that the different CCR5 epitopes are not equally exposed on cell surface receptors and among cell types.

To assess whether we could detect different antigenic forms of CCR5, as previously reported for activated CD4^+^ T cells [35, 37], we performed titration experiments with the mAbs MC5, CTC5, and 2D7 to compare their functional affinities for CCR5 in the different cellular backgrounds (Fig. 1E). Although all 3 mAbs showed comparable functional affinities for CCR5 on CHO-CCR5 cells, with 1/2 *B*_max_ values of approximately 0.55 µg/ml, significant differences were seen for CCR5-expressed on blood cells. The functional affinity determined for MC5 on MDMs and T cell blasts was comparable to that obtained on CHO-CCR5 cells. In contrast, the functional affinity of CTC5 was reduced by at least 10-fold on MDMs and T cell blasts, with binding saturation not reached at the highest concentration of antibody used (25 µg/ml; 1/2 *B*_max_ > 5.5 µg/ml). We also noted a significant reduction in the functional affinity of 2D7 on T cell blasts (1/2 *B*_max_ = 2.5 µg/ml), but not on MDMs, which suggests that T lymphocytes and myeloid cells may also differ.

### Distinguishable pools of CCR5 at the surface of human blood cells

**Figure 2.**
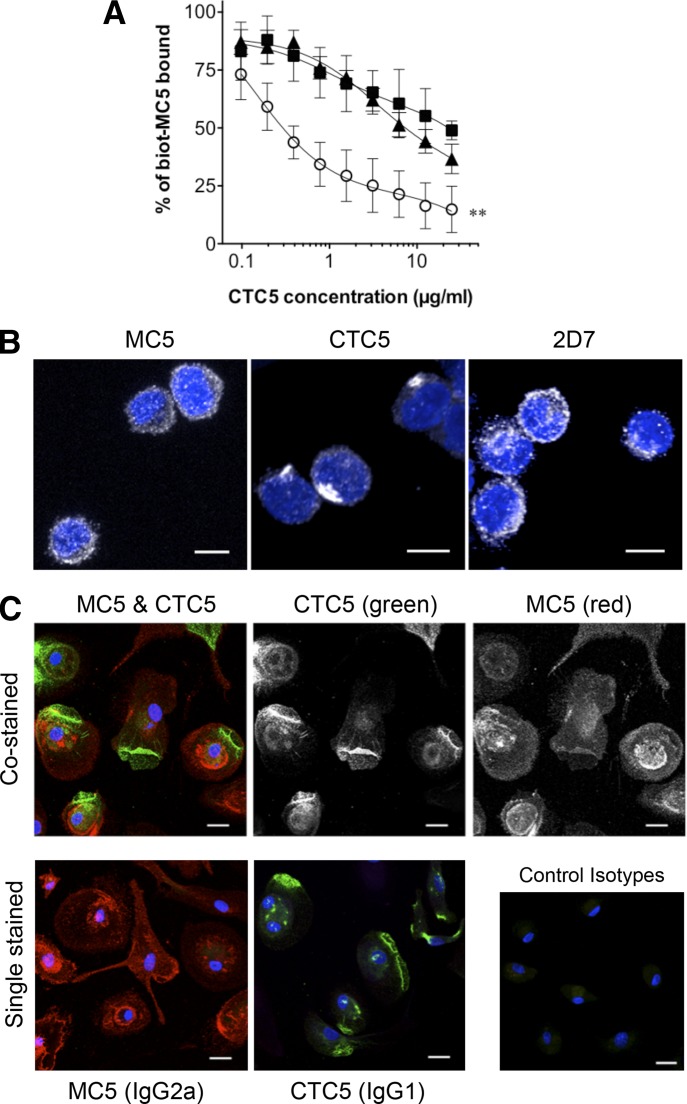
Differential CCR5 recognition by the mAbs CTC5 and MC5. (A) Binding-competition assay of biotin-MC5 on CHO-CCR5 cells (○), T-cell blasts (▴), and monocytes (▪) pretreated with increasing concentration of CTC5; results are expressed as percentages of the biotin-MC5 bound in absence of CTC5, and the graph shows the means ± sd of *N* = 4 independent, triplicate experiments. **P ≤ 0.01 one-way ANOVA and Tukey multiple comparison posttest. (B) Cell surface distribution of CCR5 on T cell blasts. Cells were stained fixed and intact with the indicated anti-CCR5 mAb (white); XY maximum-intensity projections reconstituted from the Z stack confocal images with the nuclear stain DAPI (blue). Scale bars, 5 µm. (C) Cell surface distribution of CCR5 on fixed and intact MDMs costained with MC5 and CTC5 (top panels), single stained with MC5 or CTC5, or costained with IgG1 and IgG2a control isotypes (lower panels). Cell-bound antibodies were detected with Alexa Fluor-594 anti-mouse IgG2a (red) and Alexa Fluor-488 anti-mouse IgG1 (green) secondary antibodies; XY maximum-intensity projections reconstituted from Z stack images with the nuclear stain DAPI (blue). Scale bar, 10 µm.

**Figure 3.**
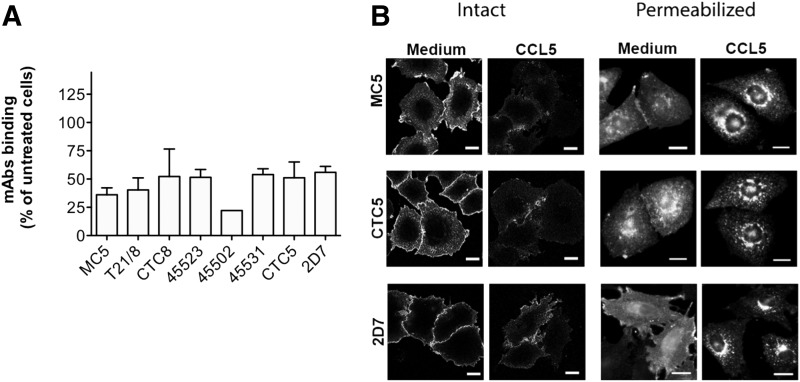
CCL5-triggered CCR5 down-modulation on CHO-CCR5 cells. (A) Cells were treated with CCL5 (100 nM) for 1 h at 37°C before samples were immunolabeled for cell surface CCR5 using the different mAbs at 5 µg/ml and analyzed by flow cytometry. Results are expressed as the percentage of staining on cells exposed to medium alone (untreated). Assays were performed in triplicate, and the graph represents the means ± sd of *N* = 3 independent experiments. (B) Immunofluorescence analysis of CCR5 distribution in CHO-CCR5 cells kept in medium or treated with CCL5 for 1 h. Samples were fixed and cells stained either intact or permeabilized to visualize cell surface or total receptors, respectively. Panels show representative single-confocal sections. Scale bar, 10 µm.

The marked change in CTC5 binding affinity between transfected and endogenously expressed CCR5 points toward different antigenic forms of the receptor. We carried out binding-competition assays between CTC5 and MC5, which recognized overlapping epitopes at the N-terminal extremity of CCR5 (Fig. 1A). Because CTC5 showed a lower binding affinity than MC5 did for CCR5 on human blood cells, we chose to perform indirect binding-competitive assays to assess how prebinding of CTC5 used at different concentration affects a subsequent cell surface binding of biotin-MC5 (5 µg/ml; **Fig. 2A**). Although CTC5 completely prevented MC5 binding to CCR5 on CHO-CCR5 cells, with 50% inhibition at 0.5 µg/ml, it was significantly less effective at blocking MC5 binding on T cell blasts and monocytes, with 50% inhibition barely achieved at the highest concentration used (30 µg/ml; Fig. 2A). Because we excluded the possibility that CTC5 binds to a structure other than CCR5 on monocytes/MDMs (Supplemental Fig. 2D and G), we hypothesized that despite the similarity of their linear epitopes, CTC5 and MC5 recognize distinct pools of CCR5-expressed on blood cells. This hypothesis was supported by immunofluorescence experiments (Fig. 2B–C), in which T cell blasts showed a “capped” labeling pattern with CTC5, contrasting with the cell surface staining seen with MC5 or 2D7 (Fig. 2B). This capped pattern was also seen with CTC5 on MDMs and contrasted with the relatively uniform pattern for MC5-bound receptors. Colabeling experiments performed on intact MDMs labeled with CTC5 and MC5 confirmed the different staining patterns of these 2 mAbs (Fig. 2C). We carried out colocalization analyses to quantify the spatial overlap of the cell surface CTC5 and MC5 epitopes, by determining the Manders’ overlap coefficients [56, 57] for CTC5 with MC5 (M1) or MC5 with CTC5 (M2) (Supplemental Fig. 3). We obtained mean values of M1 = 0.91 and M2 = 0.75 for *n* = 130 MDMs from 4 independent colabeling experiments. The high M1 coefficient indicates that CTC5 staining mainly colocalized with MC5. The lower M2 coefficient, which decreased on MDMs with a larger spread, indicates that part of the MC5 staining was truly distinct from CTC5. This analysis demonstrated that the MC5 mAb was able to recognize a subset of CCR5, which was spatially distinct from the form of CCR5 bound by CTC5, which supports the partial displacement of prebound MC5 by CTC5 described earlier for MDMs and T cell blasts (Fig. 2A). This is in contrast to CHO-CCR5 cells, where we observed full competition of binding between MC5 and CTC5, as well as similar uniform surface-staining patterns for the 3 mAbs (Figs. 2A and **3B**).

### A CCR5 population on monocytes and MDMs refractory to chemokine-mediated down-modulation

The panel of anti-CCR5 mAbs was next used in flow cytometry-based down-modulation assays [40, 46]. On CHO-CCR5 cells, the specific binding of each mAb was reduced to a similar extent (approximately 50%) following a 1 h treatment with the CCR5 ligand CCL5 (Fig. 3A). Immunofluorescence microscopy was used to confirm that the reduction was due to CCR5 internalization rather than masking of epitopes by the chemokine. CHO-CCR5 cells treated or not with CCL5 were fixed and labeled intact or permeabilized to visualize the cell surface and internal receptors, respectively (Fig. 3B). On intact cells, MC5, CTC5, and 2D7 surface staining reduced with chemokine treatment, whereas permeabilized cells showed a bright, perinuclear staining for CCL5-treated, but not untreated, cells. This confirms our previous studies and those of others, which established that CCL5 causes CCR5 down-modulation by triggering receptor endocytosis [23, 39, 45, 58].

**Figure 4.**
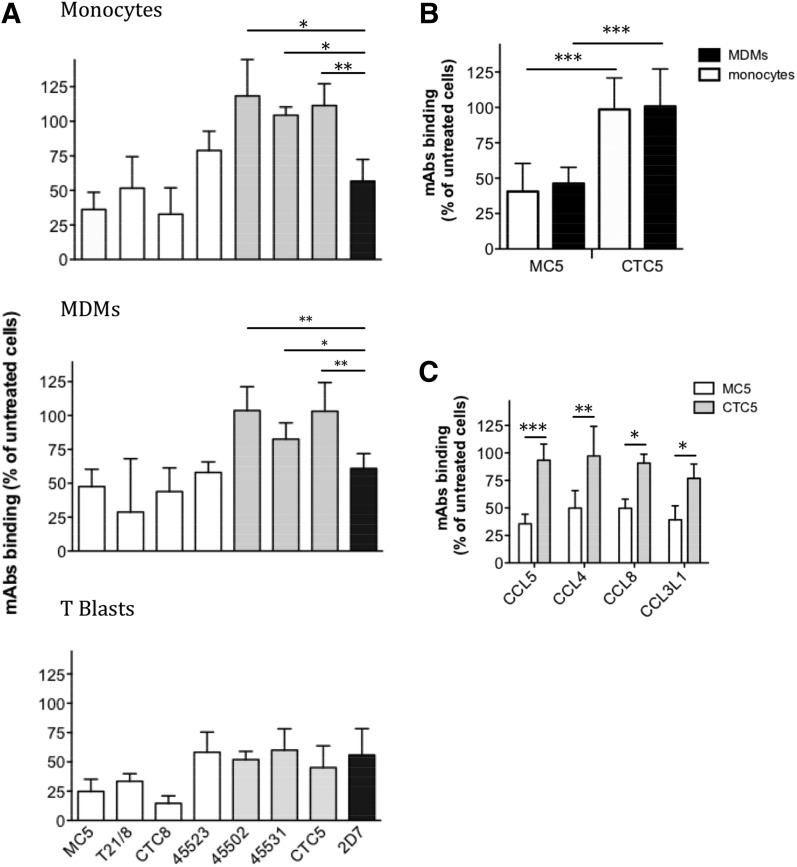
Chemokine-mediated CCR5 down-modulation on primary human blood-isolated cells. (A) Monocytes, MDMs, and T cell blasts were treated with CCL5 (100 nM) for 1 h at 37°C before samples were immunolabeled for cell surface CCR5 using the indicated mAbs at 5 µg/ml and analyzed by flow cytometry. Results are expressed as the percentage of expression on cells exposed to the medium alone (untreated) from *N* = 5 donors. Monocytes or MDMs down-modulated (white bars) and unchanged epitopes (light gray bars) are compared with 2D7 (black bar). (B) Statistical difference in cell surface levels of MC5 and CTC5 epitopes after CCL5 treatment of monocytes (*N* = 10) and MDMs (*N* = 13). (C) Cell surface levels of MC5 and CTC5 epitopes were measured after 1 h treatment of MDMs with various CCR5-binding chemokines (100 nM; *N* = 3). Each graph represents the means ± sd from experiments performed in triplicate on cells from N donors. *P ≤ 0.05. **P ≤ 0.01, ***P ≤ 0.001 by unpaired 2-tailed Student’s *t* test.

The same panel of mAbs was then used to study CCR5 down-modulation on human blood cells (**Fig. 4**). On T cell blasts, CCL5 treatment robustly reduced binding signals from all anti-CCR5 mAbs, whereas on monocytes and MDMs, only the mAbs MC5, T21/8, CTC8, 45523, and 2D7 showed reduced binding signals. For both monocytes and MDMs, binding of mAbs 45502, 45531, and CTC5 was unaffected by CCL5 treatment and was statistically different (*P* < 0.05) from 2D7 (Fig. 4A). We excluded the possibility that this unexpected observation was due to interindividual variability by repeating the experiment using mAbs MC5 and CTC5 on paired monocytes and MDMs from an additional 10 and 13 donors, respectively (Fig. 4B). We found a highly significant difference (*P* ≤ 0.001) in the binding signal obtained with MC5 compared with CTC5 after CCL5 treatment. Comparable results were obtained with several other natural chemokine ligands of CCR5, as illustrated for MDMs with CCL4, CCL8, or CCL3L1 (Fig. 4C).

**Figure 5.**
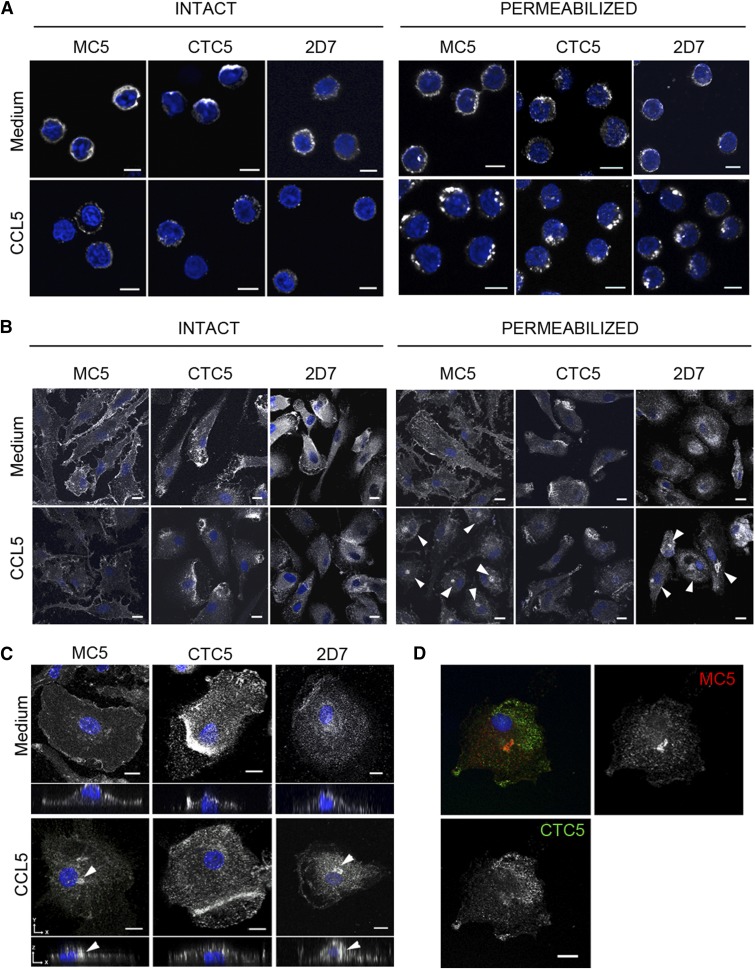
Distribution and CCL5-mediated internalization of CCR5 on human blood cells. T-cell blasts (A) and MDMs (B–D) were incubated in medium alone or with CCL5 (100 nM) for 1 h at 37°C before samples were stained with MC5, 2D7, or CTC5, as indicated. (A–B) cells were fixed and stained intact or permeabilized to visualize cell surface or total receptors, respectively. Individual panels show representative single-confocal sections depicting fields of views for T cell blasts or MDMs; arrowheads highlight the perinuclear accumulation of CCR5 in MDMs (B). (C) XY maximum-intensity projections with a XZ cross-section view reconstituted from Z-stack confocal images of permeabilized MDMs. (D) A representative maximum-intensity projection image of permeabilized, CCL5-treated MDMs costained with MC5 (red) and CTC5 (green); all samples were DAPI stained (blue). Scale bars, 5 µm (A) and 10 µm (B–D).

To further investigate this apparent difference in susceptibility to down-modulation, we used immunofluorescence confocal microscopy to analyze the cellular distribution of CCR5 labeled with the anti-CCR5 mAbs MC5, CTC5, and 2D7 following treatment with CCL5 (**Fig. 5**). T cell blasts (Fig. 5A) or MDMs (Fig. 5B), treated or not, with CCL5 for 1 h were fixed and labeled either intact or permeabilized to visualize cell surface and internal receptors. For T cell blasts, staining with all 3 antibodies was consistent with CCL5-mediated internalization of CCR5, with untreated cells showing prominent cell surface staining that was comparable for intact and permeabilized samples. Following CCL5 treatment, the surface staining on intact T cell blasts was reduced and, on permeabilized cells, was replaced by a bright vesicular staining consistent with an internal accumulation of CCR5 (Fig. 5A). On MDMs, CCR5 detected with MC5 and 2D7 showed a loss of cell surface labeling on intact cells after CCL5 treatment, which corresponded with the appearance of perinuclear staining on permeabilized cells (Fig. 5B, arrowheads), confirming ligand-mediated internalization of CCR5. However, there was no visible change in CTC5 staining following CCL5 treatment for MDMs stained either intact or permeabilized, suggesting that CCR5 receptors detected by CTC5 remain at the cell surface. Single-cell projections and cross-sections illustrate the difference among MC5 or 2D7 and CTC5 staining patterns on CCL5-treated MDMs most effectively (Fig. 5C). Resistance to ligand-mediated internalization for the CTC5-detected form of CCR5 was also seen with monocytes (Supplemental Fig. 4). The differential behavior of MC5- and CTC5-detected receptors on individual cells was confirmed by costaining permeabilized MDMs exposed to CCL5, as illustrated in Fig. 5D.

Together, our down-modulation and imaging experiments indicate that, although the populations of CCR5 recognized by all 3 mAbs tested are readily internalized by CCL5 in T cell blasts, on monocytes and MDMs, the population of CTC5-bound receptors is refractory to CCL5-mediated down-modulation.

### HIV-1 coreceptor function of CTC5-recognized CCR5 on MDMs

**Figure 6.**
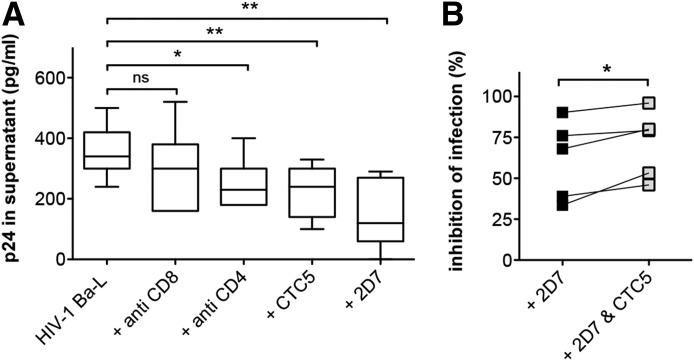
Effect of the CTC5 mAb on HIV-1 replication in MDMs. (A) MDMs, untreated or pretreated with the indicated antibodies at 12.5 µg/ml, were infected with HIV-1_Ba-L_ as described in Material and Methods. The viral production was assessed after a single-round infection, and the box and whiskers plot shows the amount of HIV-1 p24 released in culture supernatants (*N* = 7); ns, not significant. (B) Similar single-round infections showing a cooperative effect of CTC5 and 2D7 on MDMs. The graph presents the inhibition of infection compared with the infection of MDMs not pretreated with antibodies (*N* = 5). *P ≤ 0.05. **P ≤ 0.01 by paired 2-tailed Student’s *t* test.

We next tested whether the CTC5-labeled population of CCR5 on myeloid cells retained its function as an HIV-1 coreceptor. We performed mAb-inhibition studies based on single-round infection assays using HIV-1_Ba-L_ and MDMs (**Fig. 6**). Cells were pretreated with 12.5 µg/ml CTC5, positive-control mAbs known to inhibit virus entry (an anti-CD4 or 2D7), or an anti-CD8 mAb used as a control for nonspecific immunoglobulin-mediated inhibition [59]. Significant inhibition of HIV-1_Ba-L_ replication was obtained with MDMs pretreated with the positive control antibodies and CTC5 but not with the negative control anti-CD8 mAb (Fig. 6A). We assessed whether the 2D7 and CTC5 mAbs could have a synergistic inhibitory effect, by comparing HIV-1 infection of MDMs pretreated with 2D7 alone or together with CTC5. Figure 6B shows that a small but statistically significant increase in the level of inhibition was noted when CTC5 was present. Hence, although the population of CCR5 on myeloid cells that was recognized by mAb CTC5 was refractory to CCL5-mediated down-modulation, it was functional as an HIV-1 coreceptor.

### Ligand- and time-dependent down-modulation of the different antigenic forms of CCR5 on monocytes and MDMs

**Figure 7.**
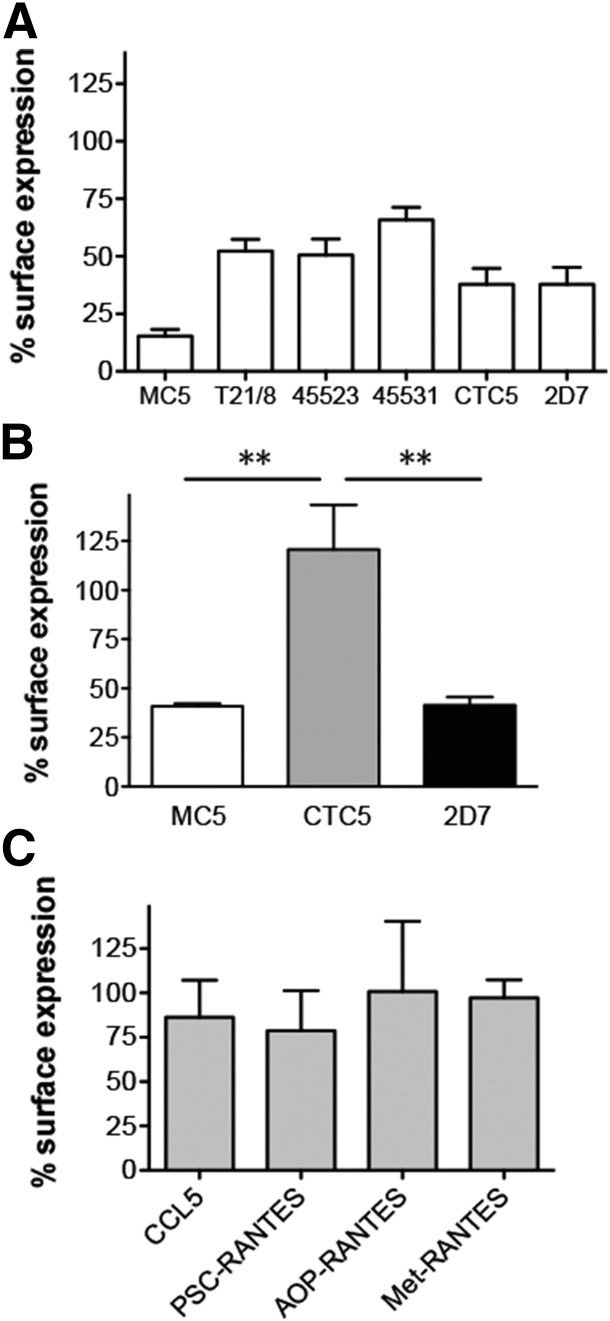
Chemokine analogs and down-modulation of CCR5 on myeloid cells. (A) T-cell blasts were treated for 1 h with PSC-RANTES to assess changes in cell surface levels of various CCR5 epitopes using the indicated antibodies (*N* = 6). (B) A similar experiment carried out on monocytes treated for 1 h with 100 nM PSC-RANTES and labeled with the indicated mAbs. (C) Detection of CTC5 cell surface levels after MDMs were treated for 1 h with CCL5 or different chemokine analogs (100 nM; *N* = 2). Graphs represent the means ± sd from independent experiments performed in triplicate on cells from *N* donors. **P ≤ 0.01 by paired 2-tailed Student’s *t* test.

Several analogs of CCL5, including PSC-RANTES, have been shown to efficiently elicit CCR5 down-modulation from the surface of transfected and primary human T cells [23, 27, 58]. In agreement with previous studies [27, 58], we found that, on T cell blasts, PSC-RANTES treatment led to a pronounced loss of cell surface CCR5 detected by all the anti-CCR5 antibodies tested (**Fig. 7A**). However, on monocytes, PSC-RANTES had a significant effect on CCR5 labeled with MC5 and 2D7 but not on CTC5 (Fig. 7B). On MDMs, PSC-RANTES as well as 2 other CCL5 analogs, amino-oxypentane (AOP)-RANTES and Met-RANTES, did not affect the CTC5 epitope after an hour of treatment (Fig. 7C).

**Figure 8.**
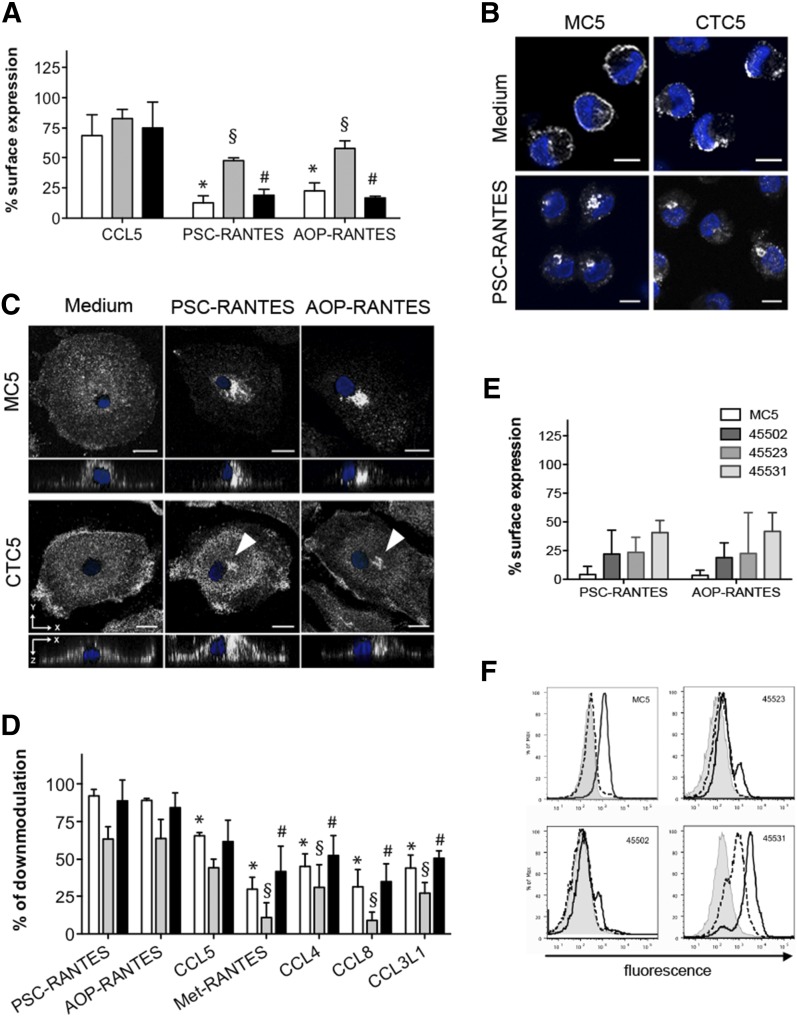
Agonist- and time-dependent effects on CTC5 epitope in myeloid cells. (A) Significant reduction of MC5 (▫), CTC5 (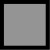), and 2D7 (▪) cell surface levels following overnight treatment of monocytes with (100 nM) PSC-RANTES or AOP-RANTES compared with CCL5; *P* < 0.05 for MC5 (*), CTC5 (§), and 2D7 (#), with 1-way ANOVA and Tukey’s multiple comparison posttest (*N* = 5). (B) Monocytes left in medium or treated overnight with PSC-RANTES were fixed, permeabilized, and stained with MC5 or CTC5. Each panel shows a representative single-confocal section. Scale bar, 5 µm. (C) CCR5 distribution in MDMs after overnight treatment followed by staining of fixed and permeabilized cells with MC5 or CTC5. Panels show XY maximum-intensity projections with an XZ cross-section view reconstituted from Z stack images. Scale bar, 10 µm. Arrowheads indicate the perinuclear compartment where superagonist-treated CCR5 accumulates. (D) Down-modulation of CCR5 on MDMs exposed overnight to a 100 nM concentration of various agonists and detected by MC5 (⬛), CTC5 (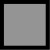), or 2D7 (▪). For each antibody, PSC-RANTES and AOP-RANTES induced significantly higher CCR5 down-modulation than other ligands. *P* < 0.05 for MC5 (*), CTC5 (§), and 2D7 (#) with 1-way ANOVA and Tukey’s multiple comparison posttest (*N* = 4). (E–F) Detection of other cell surface CCR5 epitopes on MDMs recognized by the indicated mAbs after overnight treatment with chemokine analogs (*N* = 3; E). (F) Representative histogram overlays showing the intensity of staining for each of those mAbs on untreated (bold line) or overnight AOP-RANTES-treated (dotted line) MDMs and for the relevant isotype control (gray shade) All graphs represent the means ± sd from independent experiments performed in triplicate on cells from *N* donors.

Because a previous study showed that long-term culture of human PBMCs in the presence of AOP-RANTES, but not CCL5, abolished CCR5 surface expression [23], we tested the effect of treatment duration on the surface expression of CCR5 recognized by MC5, 2D7, and CTC5 (**Fig. 8A**). Interestingly, culturing monocytes overnight with AOP-RANTES or PSC-RANTES significantly reduced the binding signal not only of MC5 and 2D7 but also of CTC5 by 50%. Consistent with this, the cellular distribution of CTC5-labeled CCR5 was modified by longer-term exposure to PSC-RANTES, with evidence of removal from the plasma membrane and accumulation in the perinuclear region of monocytes (Fig. 8B). Internal accumulation of CTC5-labeled CCR5 was also detected after long-term exposure of MDMs to either PSC- or AOP-RANTES, as illustrated in Fig. 8C. Hence, sustained exposure to strongly internalizing chemokine analogs leads to the down-modulation of CTC5-binding receptors that are refractory to internalization mediated by native chemokines.

We next compared the capacity of a panel of native CCR5 chemokines and chemokine analogs to elicit down-modulation of the antigenically distinct forms of CCR5 on MDMs with long-term treatment. Although 1 h incubation did not affect surface levels of CTC5-bound CCR5 (Figs. 4C and 7C), sustained exposure led to detectable levels of down-modulation for CTC5 with all the ligands tested, but highest (>50%) for PSC-RANTES and AOP-RANTES (Fig. 8D). MC5, CTC5, and 2D7 all showed the same trend with levels in line with the previously determined capacity of the different ligand to elicit CCR5 down-modulation [11, 27, 43].

Finally, we tested the effect of overnight treatment of MDMs with CCL5, PSC-RANTES, or AOP-RANTES on surface expression of CCR5 labeled with other anti-CCR5 mAbs, including some for which, like CTC5, no loss of surface expression was observed after an hour of incubation with CCL5 (Fig. 4A). Although some of these mAbs provided only a weak specific-binding signal on untreated MDMs (Figs. 1C and 8F), all displayed a specific reduction in cell-associated fluorescence after prolonged exposure to PSC-RANTES or AOP-RANTES (Fig. 8E–F).

Hence, the antigenically distinct form of CCR5, which is refractory to chemokine-mediated down-modulation on myeloid cells, can be removed from the cell surface, particularly by strongly internalizing chemokine analogs, but that process is significantly slower than the rapid down-modulation seen with the internalization-susceptible population of CCR5.

## DISCUSSION

GPCRs are highly conformationally flexible proteins that adopt a range of conformational states based on interactions not only with signaling ligands but also with the membrane environment and with certain cellular proteins [31, 60]. CCR5 is a GPCR, and accumulating evidence underlines the importance of allosteric modulation in its behavior, both as a chemokine receptor and as an HIV-1 coreceptor [34, 35, 37, 38, 61]. The conformational heterogeneity of CCR5 has been shown for CD4^+^ T lymphocytes, where CCR5 coupled or uncoupled to G proteins are differently used by HIV-1 strains sensitive or resistant to small molecule CCR5 inhibitors [37]. Studies on cell lines with heterologous expression of CCR5 have revealed that ligands can stabilize distinct conformations of CCR5 influenced by G protein association, β-arrestin binding, or membrane localization, which can affect receptor signaling and down-modulation [34–36, 38, 62, 63]. For these reasons, changing the cellular background in which CCR5 is expressed may have a profound effect on its functional behavior.

In our study, using a similar set of anti-CCR5 mAbs to the ones used to demonstrate conformational heterogeneity on activated CD4^+^ T cells [37], we were able to confirm this observation for T cells in general and extend it to the other major leukocyte subsets on which CCR5 is expressed, namely monocytes and macrophages.

In addition, using fluorescence microscopy, we demonstrated that the antigenically distinct forms of CCR5 exist in pools that are differentially distributed on the surface of blood-isolated cells. One population that is stained by mAbs MC5 and 2D7 has a uniform distribution, whereas the other labeled by CTC5 presents a more capped distribution (Figs. 2 and 5). Our quantitative analyses of MC5 and CTC5 colocalization at the surface of MDMs (Supplemental Fig. 3), as well as the competitive binding experiments, suggest that the pool of MC5-recognized receptors is at least partially separate from CTC5-recognized receptors.

We demonstrate that the different pools of CCR5 show differential susceptibility to cell surface down-modulation. Intriguingly, these differences are only apparent in cells of the myeloid lineage; whereas all CCR5 receptors are internalized to a similar extent on all the activated T cells tested, the capped CCR5 population recognized by CTC5, 45502, and 45531 is clearly refractory to ligand-mediated internalization on myeloid cells (Figs. 4, 5, and 7 and Supplemental Fig. 4). Interestingly, the fact that none of the samples of activated T cells used in the study showed any resistance to ligand-induced down-modulation suggests that CCR5 polymorphism is unlikely to account for the behaviors of the different pools of receptors.

The underlying molecular mechanisms responsible for the presence of distinguishable pools of CCR5 on human T cell blasts and monocytes or MDMs remain to be investigated, but there are several potential explanations for the origin of these different populations. CCR5 posttranslational modifications could account for differences like the formation of disulfide bonds between cysteine residues that link the different extracellular domains of CCR5. Mutations of these residues were shown to interfere with the activity of CCR5 as a chemokine receptor but not as a coreceptor for HIV-1 [64]. N-terminal tyrosine sulfation is also known to be important for CCR5 receptor activity [65], but is less likely to separate the populations of receptors because MC5 and CTC5 can both bind CCR5 after substitution of the tyrosine residues [47, 49]. One possibility, which has been proposed in previous work [20, 35, 37], is that differences in lipid constituents, either in different regions of a given cell or among different cell types could favor the accumulation of certain receptor conformations over others. An alternative explanation would involve interactions with cellular proteins inducing CCR5 conformations that alter antibody-binding affinity. Previous work has shown that association of cellular proteins can bias CCR5 toward binding one set of ligands rather than another and that certain ligands bias the receptor toward distinct signaling pathways [34, 63, 66]. Candidates for CCR5 conformation-inducing proteins are abundant: 1) other neighboring GPCRs [67]; 2) β-arrestins, which target ligand-bound CCR5 for internalization [40, 62, 68]; and 3) heterotrimeric G proteins, which have been shown in several recent studies to affect CCR5-binding affinity for different ligands including native chemokines, small molecule inhibitors, and the HIV envelope glycoprotein [34, 38]. These possibilities are not mutually exclusive, and different combinations of factors could contribute to the cell type- and ligand-dependent effects on CCR5 down-modulation that we report in our study. However, most findings related to conformation-inducing proteins were generated using transfected cells overexpressing CCR5 and remain to be investigated in endogenous conditions.

It is noteworthy that we show that the capped population of CCR5, which is refractory to chemokine-mediated down-modulation on myeloid cells, retains its HIV coreceptor activity. It has previously been shown that CTC5 inhibits HIV-1 entry in CCR5 transfected CD4^+^ cell lines and CD4^+^ T cells [37, 69], and we now show that this antibody reduced infection of MDMs (Fig. 6). The modest, but significant, additive effect of CTC5 to 2D7-inhibiting MDM infection supports the idea that the CCR5 forms are recognized by the 2 mAbs are partially nonoverlapping. Because CCR5 down-modulation is a key component of the anti-HIV inhibitory mechanism of native chemokines [70, 71] and chemokine analogs [26, 72], a CCR5 population that functions as a coreceptor but is refractory to chemokine-mediated down-modulation might provide a point of vulnerability for virus entry. We speculate that the presence of this potential gateway on myeloid cells explains the long-observed phenomenon that native chemokines are much less effective at protecting macrophages from CCR5-mediated HIV infection than they are at protecting CD4^+^ T cells [8, 13–18, 73].

Several chemokine analogs with potent anti-HIV activity have been shown to better protect macrophages from infection by R5-tropic HIV [23, 72, 74]. Archetypal of these analogs is AOP-RANTES, an N-terminally modified derivative of CCL5, whose enhanced antiviral activity is due to a greater capacity than native chemokines have to elicit CCR5 down-modulation, both in extent and duration [23]. In line with this observation, we found that AOP-RANTES, as well as PSC-RANTES, a more potent analog with a similar inhibitory mechanism [27, 28, 58], is capable of inducing down-modulation of the refractory capped CCR5 population on myeloid cells, albeit with slower kinetics than those observed for the permissive “uniform” population (Figs. 7 and 8). These slow kinetics may explain why a recent study, based on evidence of chemokine-mediated down-modulation after a short period of treatment, concluded that mAbs CTC5, 45502, and 45531 lack specificity for CCR5 expressed on macrophages [75]. The mechanism by which chemokine analogs achieve down-modulation of this refractory population is as yet unclear but could relate to 1) the stronger signaling activity of these molecules [76, 77], 2) the different signals elicited compared with native chemokine signaling [77], or 3) the capacity of these molecules to remain associated with CCR5 for a longer duration than the native chemokines [39, 58].

As the elucidation of the structure and molecular dynamics of CCR5 progresses [78], the importance of allosteric modulation to fine-tune its activity is becoming increasingly apparent [33]. Our work defining antigenically and functionally distinct receptor populations on different subsets of human blood cells adds to our growing knowledge of CCR5 biology, which will help to better understand the limitations of existing HIV entry inhibitors that target CCR5 and to inform strategies to improve those inhibitors.

## AUTHORSHIP

J.M.F., R.K., and N.S. performed the experiments presented in the paper. O.H. contributed to the design of experiments and the writing of the manuscript, together with N.S. who was responsible for the study.

## Supplementary Material

Supplemental Data

## Abbreviations

1/2 *B*_max_: half-maximal binding
ABS: antibody-binding sites
AOP: aminooxypentane
BM: binding medium
CCR5: CC chemokine receptor 5
CHO: Chinese hamster ovary
DHFR: dihydrofolate reductase
ECL: extracellular loop
GPCR: G protein-coupled receptor
MDMs: monocyte-derived macrophages
MFI: mean fluorescence intensity
MOI: multiplicity of infection
PSC-RANTES: Nα(n-nonanoyl)-des-Ser(1)-[l-thioprolyl(2), l-cyclohexylglycyl(3)]-RANTES(4-68))
RT: room temperature

## References

[1] Horuk R. (2001) Chemokine receptors. Cytokine Growth Factor Rev. 12, 313–335. 11544102 10.1016/s1359-6101(01)00014-4

[2] Zlotnik A., Yoshie O. (2012) The chemokine superfamily revisited. Immunity 36, 705–716. 22633458 10.1016/j.immuni.2012.05.008 PMC3396424

[3] Bennett L. D., Fox J. M., Signoret N. (2011) Mechanisms regulating chemokine receptor activity. Immunology 134, 246–256. 21977995 10.1111/j.1365-2567.2011.03485.x PMC3209565

[4] Alkhatib G., Combadiere C., Broder C. C., Feng Y., Kennedy P. E., Murphy P. M., Berger E. A. (1996) CC CKR5: a RANTES, MIP-1alpha, MIP-1beta receptor as a fusion cofactor for macrophage-tropic HIV-1. Science 272, 1955–1958. 8658171 10.1126/science.272.5270.1955

[5] Choe H., Farzan M., Sun Y., Sullivan N., Rollins B., Ponath P. D., Wu L., Mackay C. R., LaRosa G., Newman W., Gerard N., Gerard C., Sodroski J. (1996) The beta-chemokine receptors CCR3 and CCR5 facilitate infection by primary HIV-1 isolates. Cell 85, 1135–1148. 8674119 10.1016/s0092-8674(00)81313-6

[6] Deng H., Liu R., Ellmeier W., Choe S., Unutmaz D., Burkhart M., Di Marzio P., Marmon S., Sutton R. E., Hill C. M., Davis C. B., Peiper S. C., Schall T. J., Littman D. R., Landau N. R. (1996) Identification of a major co-receptor for primary isolates of HIV-1. Nature 381, 661–666. 8649511 10.1038/381661a0

[7] Doranz B. J., Rucker J., Yi Y., Smyth R. J., Samson M., Peiper S. C., Parmentier M., Collman R. G., Doms R. W. (1996) A dual-tropic primary HIV-1 isolate that uses fusin and the beta-chemokine receptors CKR-5, CKR-3, and CKR-2b as fusion cofactors. Cell 85, 1149–1158. 8674120 10.1016/s0092-8674(00)81314-8

[8] Dragic T., Litwin V., Allaway G. P., Martin S. R., Huang Y., Nagashima K. A., Cayanan C., Maddon P. J., Koup R. A., Moore J. P., Paxton W. A. (1996) HIV-1 entry into CD4^+^ cells is mediated by the chemokine receptor CC-CKR-5. Nature 381, 667–673. 8649512 10.1038/381667a0

[9] Cocchi F., DeVico A. L., Garzino-Demo A., Arya S. K., Gallo R. C., Lusso P. (1995) Identification of RANTES, MIP-1 alpha, and MIP-1 beta as the major HIV-suppressive factors produced by CD8+ T cells. Science 270, 1811–1815. 8525373 10.1126/science.270.5243.1811

[10] Scarlatti G., Tresoldi E., Björndal A., Fredriksson R., Colognesi C., Deng H. K., Malnati M. S., Plebani A., Siccardi A. G., Littman D. R., Fenyö E. M., Lusso P. (1997) In vivo evolution of HIV-1 co-receptor usage and sensitivity to chemokine-mediated suppression. Nat. Med. 3, 1259–1265. 9359702 10.1038/nm1197-1259

[11] Trkola A., Paxton W. A., Monard S. P., Hoxie J. A., Siani M. A., Thompson D. A., Wu L., Mackay C. R., Horuk R., Moore J. P. (1998) Genetic subtype-independent inhibition of human immunodeficiency virus type 1 replication by CC and CXC chemokines. J. Virol. 72, 396–404. 9420238 10.1128/jvi.72.1.396-404.1998 PMC109387

[12] Princen K., Hatse S., Vermeire K., De Clercq E., Schols D. (2004) Establishment of a novel CCR5 and CXCR4 expressing CD4^+^ cell line which is highly sensitive to HIV and suitable for high-throughput evaluation of CCR5 and CXCR4 antagonists. Retrovirology 1, 2. 15169555 10.1186/1742-4690-1-2 PMC416571

[13] Schmidtmayerova H., Sherry B., Bukrinsky M. (1996) Chemokines and HIV replication. Nature 382, 767. 8752270 10.1038/382767a0

[14] Moriuchi H., Moriuchi M., Combadiere C., Murphy P. M., Fauci A. S. (1996) CD8^+^ T-cell-derived soluble factor(s), but not β-chemokines RANTES, MIP-1α, and MIP-1β, suppress HIV-1 replication in monocyte/macrophages. Proc. Natl. Acad. Sci. USA 93, 15341–15345. 8986813 10.1073/pnas.93.26.15341 PMC26406

[15] Oravecz T., Pall M., Wang J., Roderiquez G., Ditto M., Norcross M. A. (1997) Regulation of anti-HIV-1 activity of RANTES by heparan sulfate proteoglycans. J. Immunol. 159, 4587–4592. 9379060

[16] Aquaro S., Menten P., Struyf S., Proost P., Van Damme J., De Clercq E., Schols D. (2001) The LD78beta isoform of MIP-1α is the most potent CC-chemokine in inhibiting CCR5-dependent human immunodeficiency virus type 1 replication in human macrophages. J. Virol. 75, 4402–4406. 11287590 10.1128/JVI.75.9.4402-4406.2001 PMC114186

[17] Trkola A., Ketas T. J., Nagashima K. A., Zhao L., Cilliers T., Morris L., Moore J. P., Maddon P. J., Olson W. C. (2001) Potent, broad-spectrum inhibition of human immunodeficiency virus type 1 by the CCR5 monoclonal antibody PRO 140. J. Virol. 75, 579–588. 11134270 10.1128/JVI.75.2.579-588.2001 PMC113953

[18] Gross E., Amella C. A., Pompucci L., Franchin G., Sherry B., Schmidtmayerova H. (2003) Macrophages and lymphocytes differentially modulate the ability of RANTES to inhibit HIV-1 infection. J. Leukoc. Biol. 74, 781–790. 12960233 10.1189/jlb.0403187

[19] Wahl S. M., Greenwell-Wild T., Vázquez N. (2006) HIV accomplices and adversaries in macrophage infection. J. Leukoc. Biol. 80, 973–983. 16908514 10.1189/jlb.0306130

[20] Carter G. C., Bernstone L., Sangani D., Bee J. W., Harder T., James W. (2009) HIV entry in macrophages is dependent on intact lipid rafts. Virology 386, 192–202. 19185899 10.1016/j.virol.2008.12.031 PMC7103383

[21] Sterjovski J., Roche M., Churchill M. J., Ellett A., Farrugia W., Gray L. R., Cowley D., Poumbourios P., Lee B., Wesselingh S. L., Cunningham A. L., Ramsland P. A., Gorry P. R. (2010) An altered and more efficient mechanism of CCR5 engagement contributes to macrophage tropism of CCR5-using HIV-1 envelopes. Virology 404, 269–278. 20570309 10.1016/j.virol.2010.05.006 PMC3096480

[22] Cashin K., Roche M., Sterjovski J., Ellett A., Gray L. R., Cunningham A. L., Ramsland P. A., Churchill M. J., Gorry P. R. (2011) Alternative coreceptor requirements for efficient CCR5- and CXCR4-mediated HIV-1 entry into macrophages. J. Virol. 85, 10699–10709. 21835796 10.1128/JVI.05510-11 PMC3187472

[23] Mack M., Luckow B., Nelson P. J., Cihak J., Simmons G., Clapham P. R., Signoret N., Marsh M., Stangassinger M., Borlat F., Wells T. N., Schlöndorff D., Proudfoot A. E. (1998) Aminooxypentane-RANTES induces CCR5 internalization but inhibits recycling: a novel inhibitory mechanism of HIV infectivity. J. Exp. Med. 187, 1215–1224. 9547333 10.1084/jem.187.8.1215 PMC2212227

[24] Brandt S. M., Mariani R., Holland A. U., Hope T. J., Landau N. R. (2002) Association of chemokine-mediated block to HIV entry with coreceptor internalization. J. Biol. Chem. 277, 17291–17299. 11782464 10.1074/jbc.M108232200

[25] Lin Y. L., Mettling C., Portalès P., Rouzier R., Clot J., Reynes J., Corbeau P. (2008) The chemokine CCL5 regulates the in vivo cell surface expression of its receptor, CCR5. AIDS 22, 430–432. 18195571 10.1097/QAD.0b013e3282f46a6f

[26] Sabbe R., Picchio G. R., Pastore C., Chaloin O., Hartley O., Offord R., Mosier D. E. (2001) Donor- and ligand-dependent differences in C-C chemokine receptor 5 reexpression. J. Virol. 75, 661–671. 11134280 10.1128/JVI.75.2.661-671.2001 PMC113963

[27] Pastore C., Picchio G. R., Galimi F., Fish R., Hartley O., Offord R. E., Mosier D. E. (2003) Two mechanisms for human immunodeficiency virus type 1 inhibition by N-terminal modifications of RANTES. Antimicrob. Agents Chemother. 47, 509–517. 12543651 10.1128/AAC.47.2.509-517.2003 PMC151767

[28] Gaertner H., Cerini F., Escola J. M., Kuenzi G., Melotti A., Offord R., Rossitto-Borlat I., Nedellec R., Salkowitz J., Gorochov G., Mosier D., Hartley O. (2008) Highly potent, fully recombinant anti-HIV chemokines: reengineering a low-cost microbicide. Proc. Natl. Acad. Sci. U. S. A. 105, 17706–17711. 19004761 10.1073/pnas.0805098105 PMC2584686

[29] Lederman M. M., Veazey R. S., Offord R., Mosier D. E., Dufour J., Mefford M., Piatak M. Jr., Lifson J. D., Salkowitz J. R., Rodriguez B., Blauvelt A., Hartley O. (2004) Prevention of vaginal SHIV transmission in rhesus macaques through inhibition of CCR5. Science 306, 485–487. 15486300 10.1126/science.1099288

[30] Vauquelin G., Van Liefde I. (2005) G protein-coupled receptors: a count of 1001 conformations. Fundam. Clin. Pharmacol. 19, 45–56. 15660959 10.1111/j.1472-8206.2005.00319.x

[31] Park P. S.-H. (2012) Ensemble of G protein-coupled receptor active states. Curr. Med. Chem. 19, 1146–1154. 22300048 10.2174/092986712799320619 PMC3311957

[32] Wang L., Martin B., Brenneman R., Luttrell L. M., Maudsley S. (2009) Allosteric modulators of g protein-coupled receptors: future therapeutics for complex physiological disorders. J. Pharmacol. Exp. Ther. 331, 340–348. 19667132 10.1124/jpet.109.156380 PMC2775272

[33] Luttrell L. M., Kenakin T. P. (2011) Refining efficacy: allosterism and bias in G protein-coupled receptor signaling. Methods Mol. Biol. 756, 3–35. 21870218 10.1007/978-1-61779-160-4_1

[34] Colin P., Bénureau Y., Staropoli I., Wang Y., Gonzalez N., Alcami J., Hartley O., Brelot A., Arenzana-Seisdedos F., Lagane B. (2013) HIV-1 exploits CCR5 conformational heterogeneity to escape inhibition by chemokines. Proc. Natl. Acad. Sci. U. S. A. 110, 9475–9480. 23696662 10.1073/pnas.1222205110 PMC3677469

[35] Flegler A. J., Cianci G. C., Hope T. J. (2014) CCR5 conformations are dynamic and modulated by localization, trafficking and G protein association. PLoS One 9, e89056. 24586501 10.1371/journal.pone.0089056 PMC3938464

[36] Abrol R., Trzaskowski B., Goddard W. A. III, Nesterov A., Olave I., Irons C. (2014) Ligand- and mutation-induced conformational selection in the CCR5 chemokine G protein-coupled receptor. Proc. Natl. Acad. Sci. U. S. A. 111, 13040–13045. 25157173 10.1073/pnas.1413216111 PMC4246978

[37] Berro R., Klasse P. J., Lascano D., Flegler A., Nagashima K. A., Sanders R. W., Sakmar T. P., Hope T. J., Moore J. P. (2011) Multiple CCR5 conformations on the cell surface are used differentially by human immunodeficiency viruses resistant or sensitive to CCR5 inhibitors. J. Virol. 85, 8227–8240. 21680525 10.1128/JVI.00767-11 PMC3147974

[38] Berro R., Yasmeen A., Abrol R., Trzaskowski B., Abi-Habib S., Grunbeck A., Lascano D., Goddard W. A. III, Klasse P. J., Sakmar T. P., Moore J. P. (2013) Use of G-protein-coupled and -uncoupled CCR5 receptors by CCR5 inhibitor-resistant and -sensitive human immunodeficiency virus type 1 variants. J. Virol. 87, 6569–6581. 23468486 10.1128/JVI.00099-13 PMC3676082

[39] Signoret N., Pelchen-Matthews A., Mack M., Proudfoot A. E., Marsh M. (2000) Endocytosis and recycling of the HIV coreceptor CCR5. J. Cell Biol. 151, 1281–1294. 11121442 10.1083/jcb.151.6.1281 PMC2190598

[40] Fox J. M., Letellier E., Oliphant C. J., Signoret N. (2011) TLR2-dependent pathway of heterologous down-modulation for the CC chemokine receptors 1, 2, and 5 in human blood monocytes. Blood 117, 1851–1860. 21148810 10.1182/blood-2010-05-287474

[41] Proudfoot A. E., Power C. A., Hoogewerf A. J., Montjovent M. O., Borlat F., Offord R. E., Wells T. N. (1996) Extension of recombinant human RANTES by the retention of the initiating methionine produces a potent antagonist. J. Biol. Chem. 271, 2599–2603. 8576227 10.1074/jbc.271.5.2599

[42] Wilken J., Hoover D., Thompson D. A., Barlow P. N., McSparron H., Picard L., Wlodawer A., Lubkowski J., Kent S. B. (1999) Total chemical synthesis and high-resolution crystal structure of the potent anti-HIV protein AOP-RANTES. Chem. Biol. 6, 43–51. 9889151 10.1016/S1074-5521(99)80019-2

[43] Hartley O., Gaertner H., Wilken J., Thompson D., Fish R., Ramos A., Pastore C., Dufour B., Cerini F., Melotti A., Heveker N., Picard L., Alizon M., Mosier D., Kent S., Offord R. (2004) Medicinal chemistry applied to a synthetic protein: development of highly potent HIV entry inhibitors. Proc. Natl. Acad. Sci. U. S. A. 101, 16460–16465. 15545608 10.1073/pnas.0404802101 PMC534511

[44] Gartner S., Markovits P., Markovitz D. M., Kaplan M. H., Gallo R. C., Popovic M. (1986) The role of mononuclear phagocytes in HTLV-III/LAV infection. Science 233, 215–219. 3014648 10.1126/science.3014648

[45] Signoret N., Hewlett L., Wavre S., Pelchen-Matthews A., Oppermann M., Marsh M. (2005) Agonist-induced endocytosis of CC chemokine receptor 5 is clathrin dependent. Mol. Biol. Cell 16, 902–917. 15591129 10.1091/mbc.E04-08-0687 PMC545921

[46] Kershaw T., Wavre-Shapton S. T., Signoret N., Marsh M. (2009) Analysis of chemokine receptor endocytosis and intracellular trafficking. Methods Enzymol. 460, 357–377. 19446735 10.1016/S0076-6879(09)05218-5

[47] Blanpain C., Vanderwinden J. M., Cihak J., Wittamer V., Le Poul E., Issafras H., Stangassinger M., Vassart G., Marullo S., Schlndorff D., Parmentier M., Mack M. (2002) Multiple active states and oligomerization of CCR5 revealed by functional properties of monoclonal antibodies. Mol. Biol. Cell 13, 723–737. 11854425 10.1091/mbc.01-03-0129 PMC65662

[48] Blanpain C., Lee B., Tackoen M., Puffer B., Boom A., Libert F., Sharron M., Wittamer V., Vassart G., Doms R. W., Parmentier M. (2000) Multiple nonfunctional alleles of CCR5 are frequent in various human populations. Blood 96, 1638–1645. 10961858

[49] Shimizu N., Tanaka A., Oue A., Mori T., Apichartpiyakul C., Hoshino H. (2008) A short amino acid sequence containing tyrosine in the N-terminal region of G protein-coupled receptors is critical for their potential use as co-receptors for human and simian immunodeficiency viruses. J. Gen. Virol. 89, 3126–3136. 19008402 10.1099/vir.0.2008/002188-0

[50] Wu L., LaRosa G., Kassam N., Gordon C. J., Heath H., Ruffing N., Chen H., Humblias J., Samson M., Parmentier M., Moore J. P., Mackay C. R. (1997) Interaction of chemokine receptor CCR5 with its ligands: multiple domains for HIV-1 gp120 binding and a single domain for chemokine binding. J. Exp. Med. 186, 1373–1381. 9334377 10.1084/jem.186.8.1373 PMC2199098

[51] Lee B., Sharron M., Blanpain C., Doranz B. J., Vakili J., Setoh P., Berg E., Liu G., Guy H. R., Durell S. R., Parmentier M., Chang C. N., Price K., Tsang M., Doms R. W. (1999) Epitope mapping of CCR5 reveals multiple conformational states and distinct but overlapping structures involved in chemokine and coreceptor function. J. Biol. Chem. 274, 9617–9626. 10092648 10.1074/jbc.274.14.9617

[52] Lee B., Sharron M., Montaner L. J., Weissman D., Doms R. W. (1999) Quantification of CD4, CCR5, and CXCR4 levels on lymphocyte subsets, dendritic cells, and differentially conditioned monocyte-derived macrophages. Proc. Natl. Acad. Sci. U. S. A. 96, 5215–5220. 10220446 10.1073/pnas.96.9.5215 PMC21844

[53] O’Brien S. J., Moore J. P. (2000) The effect of genetic variation in chemokines and their receptors on HIV transmission and progression to AIDS. Immunol. Rev. 177, 99–111. 11138790 10.1034/j.1600-065x.2000.17710.x

[54] Tuttle D. L., Harrison J. K., Anders C., Sleasman J. W., Goodenow M. M. (1998) Expression of CCR5 increases during monocyte differentiation and directly mediates macrophage susceptibility to infection by human immunodeficiency virus type 1. J. Virol. 72, 4962–4969. 9573265 10.1128/jvi.72.6.4962-4969.1998 PMC110058

[55] Kaufmann A., Salentin R., Gemsa D., Sprenger H. (2001) Increase of CCR1 and CCR5 expression and enhanced functional response to MIP-1 alpha during differentiation of human monocytes to macrophages. J. Leukoc. Biol. 69, 248–252. 11272275

[56] Dunn K. W., Kamocka M. M., McDonald J. H. (2011) A practical guide to evaluating colocalization in biological microscopy. Am. J. Physiol. Cell Physiol. 300, C723–C742. 21209361 10.1152/ajpcell.00462.2010 PMC3074624

[57] Barlow A. L., Macleod A., Noppen S., Sanderson J., Guérin C. J. (2010) Colocalization analysis in fluorescence micrographs: verification of a more accurate calculation of Pearson’s correlation coefficient. Microsc. Microanal. 16, 710–724. 20946701 10.1017/S143192761009389X

[58] Escola J. M., Kuenzi G., Gaertner H., Foti M., Hartley O. (2010) CC chemokine receptor 5 (CCR5) desensitization: cycling receptors accumulate in the trans-Golgi network. J. Biol. Chem. 285, 41772–41780. 21041313 10.1074/jbc.M110.153460 PMC3009905

[59] Perez-Bercoff D., David A., Sudry H., Barré-Sinoussi F., Pancino G. (2003) Fcγ receptor-mediated suppression of human immunodeficiency virus type 1 replication in primary human macrophages. J. Virol. 77, 4081–4094. 12634367 10.1128/JVI.77.7.4081-4094.2003 PMC150663

[60] Nygaard R., Zou Y., Dror R. O., Mildorf T. J., Arlow D. H., Manglik A., Pan A. C., Liu C. W., Fung J. J., Bokoch M. P., Thian F. S., Kobilka T. S., Shaw D. E., Mueller L., Prosser R. S., Kobilka B. K. (2013) The dynamic process of β_2_-adrenergic receptor activation. Cell 152, 532–542. 23374348 10.1016/j.cell.2013.01.008 PMC3586676

[61] de Voux A., Chan M. C., Folefoc A. T., Madziva M. T., Flanagan C. A. (2013) Constitutively active CCR5 chemokine receptors differ in mediating HIV envelope-dependent fusion. PLoS One 8, e54532. 23355876 10.1371/journal.pone.0054532 PMC3552960

[62] Jin J., Colin P., Staropoli I., Lima-Fernandes E., Ferret C., Demir A., Rogée S., Hartley O., Randriamampita C., Scott M. G., Marullo S., Sauvonnet N., Arenzana-Seisdedos F., Lagane B., Brelot A. (2014) Targeting spare CC chemokine receptor 5 (CCR5) as a principle to inhibit HIV-1 entry. J. Biol. Chem. 289, 19042–19052. 24855645 10.1074/jbc.M114.559831 PMC4081942

[63] Corbisier J., Galès C., Huszagh A., Parmentier M., Springael J. Y. (2015) Biased signaling at chemokine receptors. J. Biol. Chem. 10.1074/jbc.M114.596098 PMC4392259 25614627

[64] Blanpain C., Lee B., Vakili J., Doranz B. J., Govaerts C., Migeotte I., Sharron M., Dupriez V., Vassart G., Doms R. W., Parmentier M. (1999) Extracellular cysteines of CCR5 are required for chemokine binding, but dispensable for HIV-1 coreceptor activity. J. Biol. Chem. 274, 18902–18908. 10383387 10.1074/jbc.274.27.18902

[65] Farzan M., Mirzabekov T., Kolchinsky P., Wyatt R., Cayabyab M., Gerard N. P., Gerard C., Sodroski J., Choe H. (1999) Tyrosine sulfation of the amino terminus of CCR5 facilitates HIV-1 entry. Cell 96, 667–676. 10089882 10.1016/s0092-8674(00)80577-2

[66] Steen A., Thiele S., Guo D., Hansen L. S., Frimurer T. M., Rosenkilde M. M. (2013) Biased and constitutive signaling in the CC-chemokine receptor CCR5 by manipulating the interface between transmembrane helices 6 and 7. J. Biol. Chem. 288, 12511–12521. 23493400 10.1074/jbc.M112.449587 PMC3642299

[67] Isik N., Hereld D., Jin T. (2008) Fluorescence resonance energy transfer imaging reveals that chemokine-binding modulates heterodimers of CXCR4 and CCR5 receptors. PLoS One 3, e3424. 18923649 10.1371/journal.pone.0003424 PMC2566588

[68] Fraile-Ramos A., Kohout T. A., Waldhoer M., Marsh M. (2003) Endocytosis of the viral chemokine receptor US28 does not require beta-arrestins but is dependent on the clathrin-mediated pathway. Traffic 4, 243–253. 12694563 10.1034/j.1600-0854.2003.00079.x

[69] Henrich T. J., Lewine N. R., Lee S. H., Rao S. S., Berro R., Gulick R. M., Moore J. P., Tsibris A. M., Kuritzkes D. R. (2012) Differential use of CCR5 by HIV-1 clinical isolates resistant to small-molecule CCR5 antagonists. Antimicrob. Agents Chemother. 56, 1931–1935. 22252820 10.1128/AAC.06061-11 PMC3318367

[70] Alkhatib G., Locati M., Kennedy P. E., Murphy P. M., Berger E. A. (1997) HIV-1 coreceptor activity of CCR5 and its inhibition by chemokines: independence from G protein signaling and importance of coreceptor downmodulation. Virology 234, 340–348. 9268166 10.1006/viro.1997.8673

[71] Aramori I., Ferguson S. S., Bieniasz P. D., Zhang J., Cullen B., Cullen M. G. (1997) Molecular mechanism of desensitization of the chemokine receptor CCR-5: receptor signaling and internalization are dissociable from its role as an HIV-1 co-receptor. EMBO J. 16, 4606–4616. 9303305 10.1093/emboj/16.15.4606 PMC1170087

[72] Simmons G., Clapham P. R., Picard L., Offord R. E., Rosenkilde M. M., Schwartz T. W., Buser R., Wells T. N. C., Proudfoot A. E. I. (1997) Potent inhibition of HIV-1 infectivity in macrophages and lymphocytes by a novel CCR5 antagonist. Science 276, 276–279. 9092481 10.1126/science.276.5310.276

[73] Ylisastigui L., Amzazi S., Bakri Y., Vizzavona J., Vita C., Gluckman J. C., Benjouad A. (1998) Effect of RANTES on the infection of monocyte-derived primary macrophages by human immunodeficiency virus type 1 and type 2. Biomed. Pharmacother. 52, 447–453. 9921414 10.1016/s0753-3322(99)80023-7

[74] Hartley O., Dorgham K., Perez-Bercoff D., Cerini F., Heimann A., Gaertner H., Offord R. E., Pancino G., Debré P., Gorochov G. (2003) Human immunodeficiency virus type 1 entry inhibitors selected on living cells from a library of phage chemokines. J. Virol. 77, 6637–6644. 12767983 10.1128/JVI.77.12.6637-6644.2003 PMC156188

[75] Bernstone L., van Wilgenburg B., James W. (2012) Several commercially available anti-CCR5 monoclonal antibodies lack specificity and should be used with caution. Hybridoma (Larchmt) 31, 7–19. 22316480 10.1089/hyb.2010.0092

[76] Vila-Coro A. J., Mellado M., Martín de Ana A., Martínez-A C., Rodríguez-Frade J. M. (1999) Characterization of RANTES- and aminooxypentane-RANTES-triggered desensitization signals reveals differences in recruitment of the G protein-coupled receptor complex. J. Immunol. 163, 3037–3044. 10477567

[77] Rodríguez-Frade J. M., Vila-Coro A. J., Martín A., Nieto M., Sánchez-Madrid F., Proudfoot A. E., Wells T. N., Martínez-A C., Mellado M. (1999) Similarities and differences in RANTES- and (AOP)-RANTES-triggered signals: implications for chemotaxis. J. Cell Biol. 144, 755–765. 10037796 10.1083/jcb.144.4.755 PMC2132943

[78] Tan Q., Zhu Y., Li J., Chen Z., Han G. W., Kufareva I., Li T., Ma L., Fenalti G., Li J., Zhang W., Xie X., Yang H., Jiang H., Cherezov V., Liu H., Stevens R. C., Zhao Q., Wu B. (2013) Structure of the CCR5 chemokine receptor-HIV entry inhibitor maraviroc complex. Science 341, 1387–1390. 24030490 10.1126/science.1241475 PMC3819204

