# Supplementary material for: CCR5 susceptibility to ligand-mediated down-modulation differs between human T lymphocytes and myeloid cells
Source: J Leukoc Biol. 2015 May 8;98(1):59–71. doi: 10.1189/jlb.2A0414-193RR (PMC4560160; doi:10.1189/jlb.2A0414-193RR)
Supplement: Supplemental Data [file supp_98_1_59__index.html]

CCR5 susceptibility to ligand-mediated down-modulation differs between human T lymphocytes and myeloid cells — CCR5 susceptibility to ligand-mediated down-modulation differs between human T lymphocytes and myeloid cells — Supplemental Data 

# CCR5 susceptibility to ligand-mediated down-modulation differs between human T lymphocytes and myeloid cells

## Supplemental Data

- Supplemental Data
- Supplemental Data
- Supplemental Data
- Supplemental Data
