# Supplementary figures and images for: CCR5 susceptibility to ligand-mediated down-modulation differs between human T lymphocytes and myeloid cells
Source: J Leukoc Biol. 2015 May 8;98(1):59–71. doi: 10.1189/jlb.2A0414-193RR (PMC4560160; doi:10.1189/jlb.2A0414-193RR)

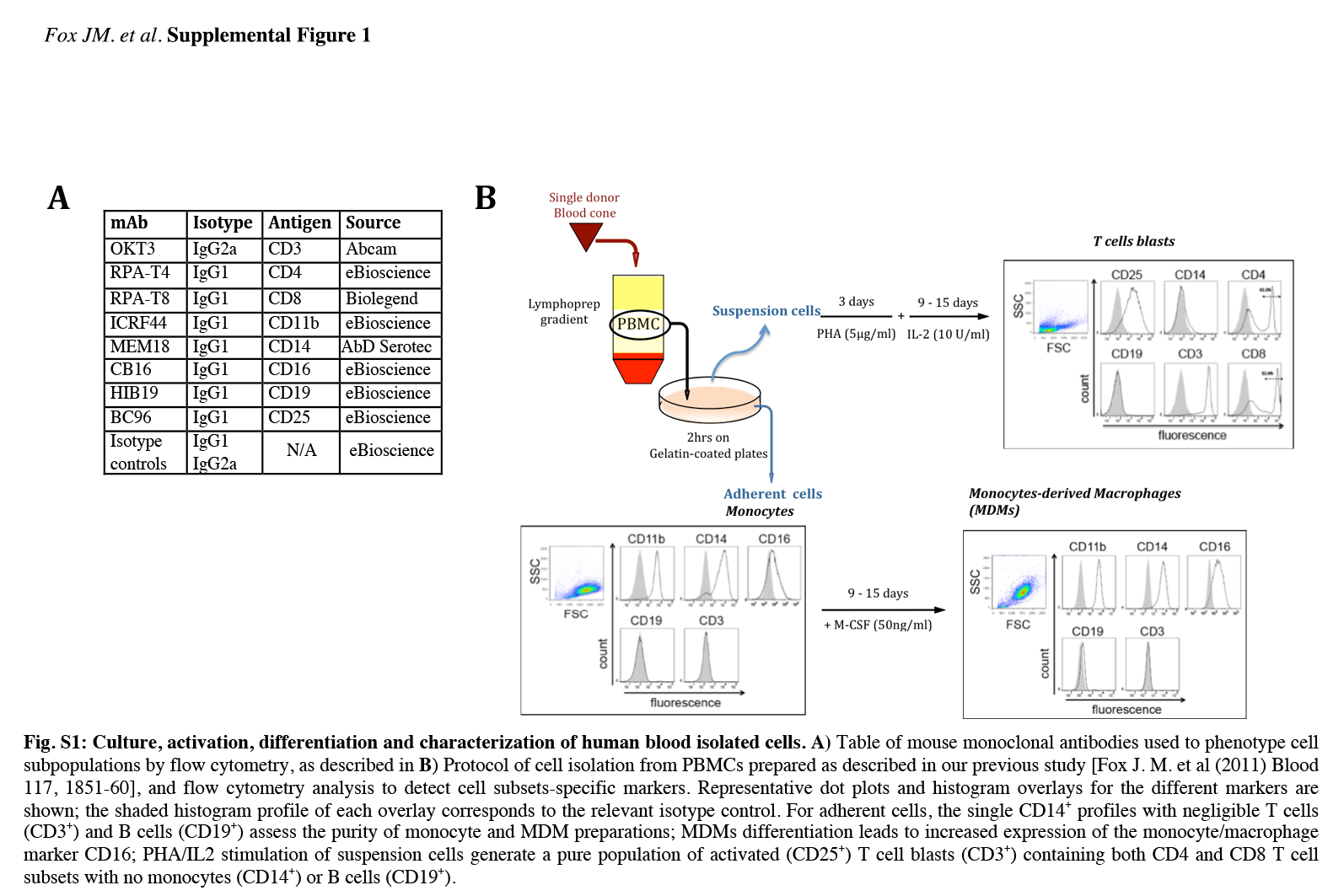

Supplement: Supplemental Data [file supp_jlb.2A0414-193RR_Supplemental_Figure1.tif]

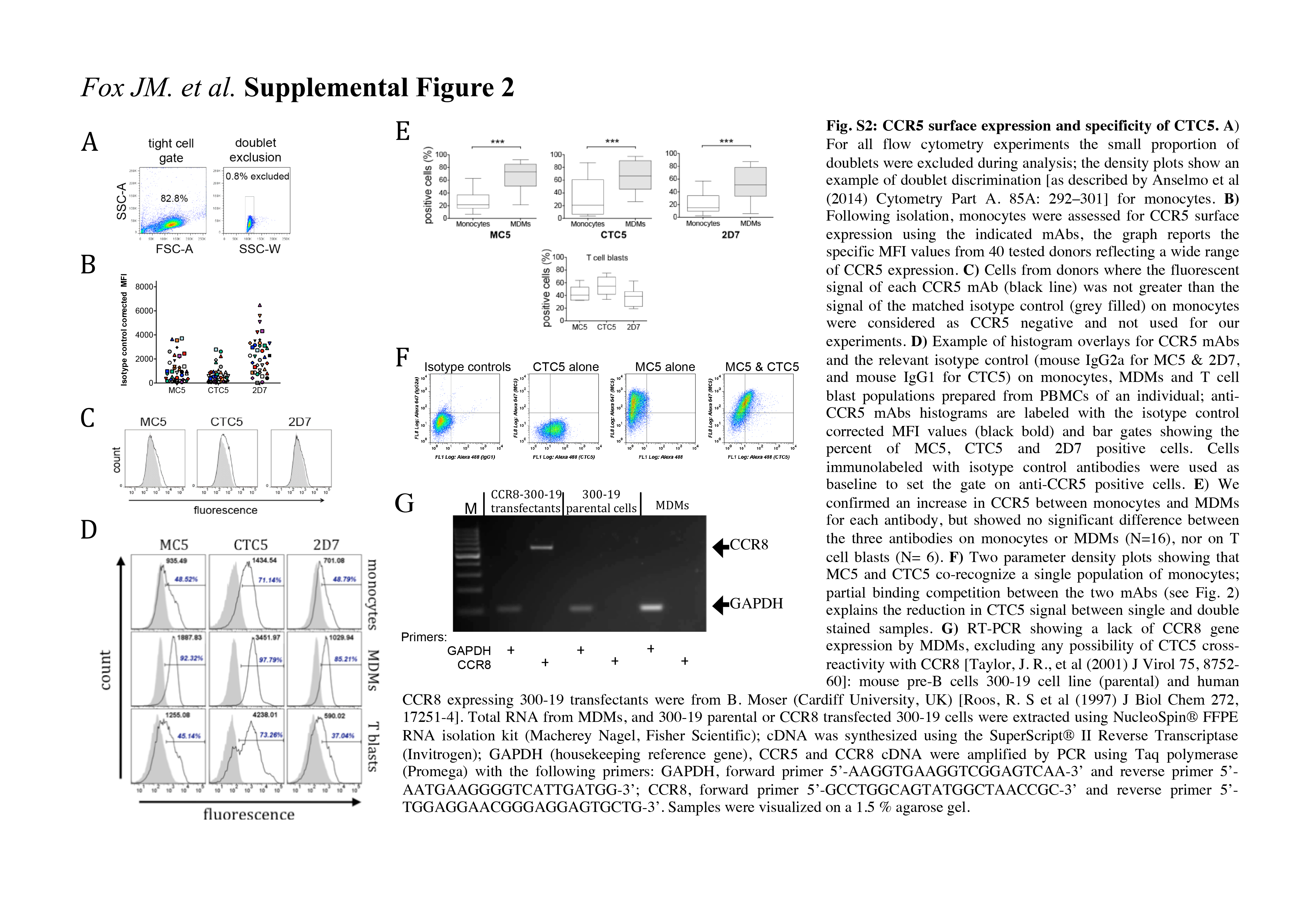

Supplement: Supplemental Data [file supp_jlb.2A0414-193RR_Supplemental_Figure2.tif]

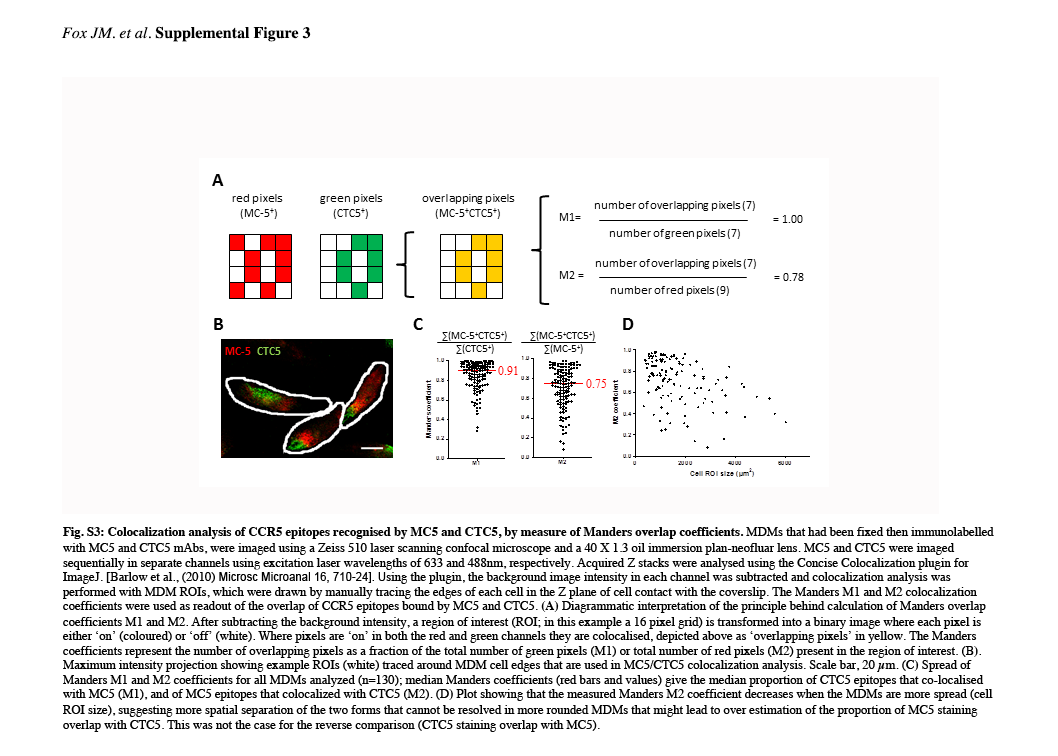

Supplement: Supplemental Data [file supp_jlb.2A0414-193RR_Supplemental_Figure3.tif]

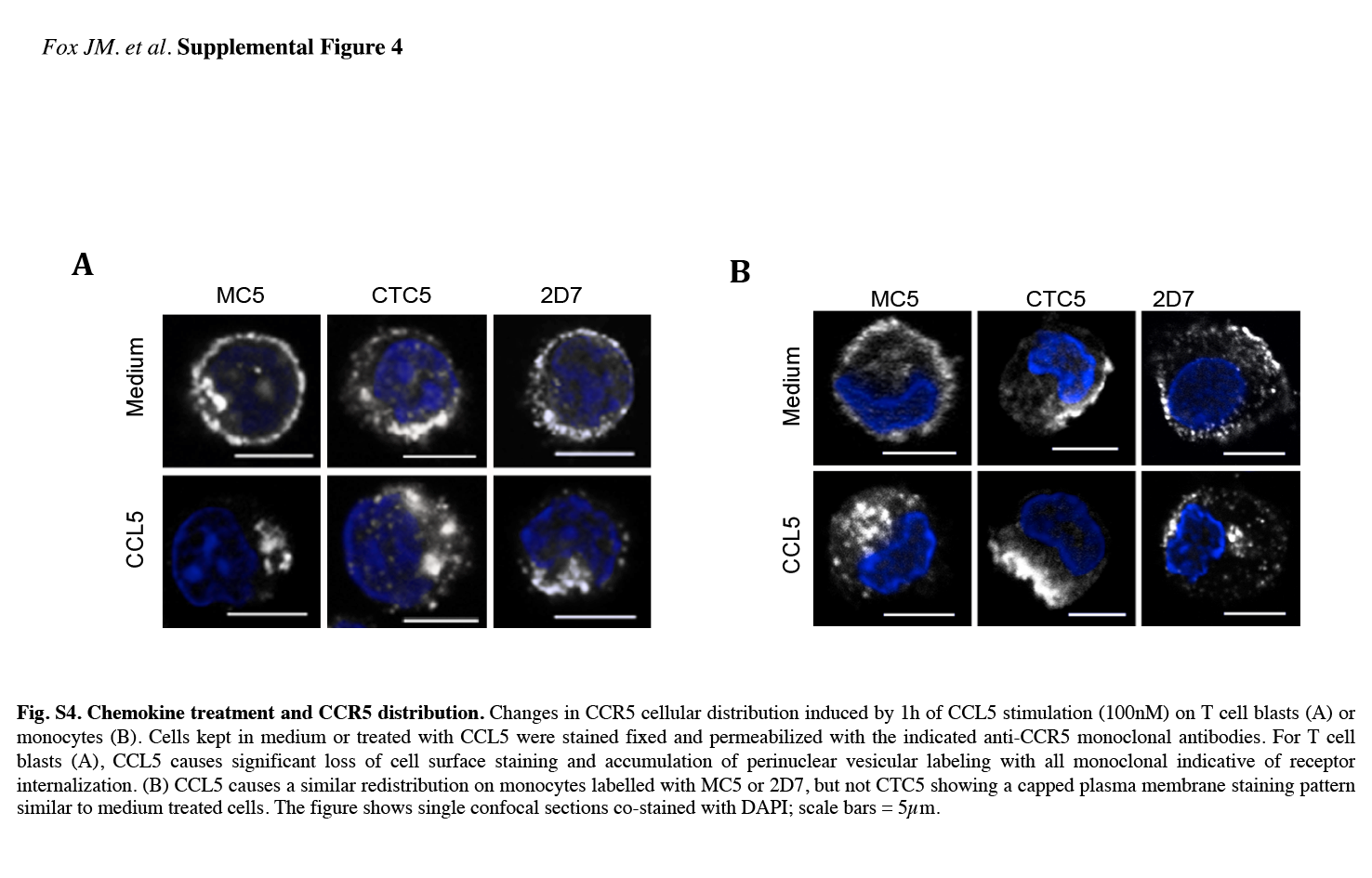

Supplement: Supplemental Data [file supp_jlb.2A0414-193RR_Supplemental_Figure4.tif]
